# Further delineation of the *SCAF4*-associated neurodevelopmental disorder

**DOI:** 10.1038/s41431-024-01760-2

**Published:** 2024-12-12

**Authors:** Cosima M. Schmid, Anne Gregor, Anna Ruiz, Carmen Manso Bazús, Isabella Herman, Farah Ammouri, Urania Kotzaeridou, Vanda McNiven, Lucie Dupuis, Katharina Steindl, Anaïs Begemann, Anita Rauch, Aude-Annick Suter, Bertrand Isidor, Sandra Mercier, Mathilde Nizon, Benjamin Cogné, Wallid Deb, Thomas Besnard, Tobias B. Haack, Ruth J. Falb, Amelie J. Müller, Tobias Linden, Chad R. Haldeman-Englert, Charlotte W. Ockeloen, Francesca Mattioli, Alexandre Reymond, Nazia Ibrahim, Shagufta Naz, Elodie Lacaze, Jennifer A. Bassetti, Julia Hoefele, Theresa Brunet, Korbinian M. Riedhammer, Houda Z. Elloumi, Richard Person, Fanggeng Zou, Juliette J. Kahle, Kirsten Cremer, Axel Schmidt, Marie-Ange Delrue, Pedro M. Almeida, Fabiana Ramos, Siddharth Srivastava, Aisling Quinlan, Stephen Robertson, Eva Manka, Alma Kuechler, Stephanie Spranger, Malgorzata J. M. Nowaczyk, Reem M. Elshafie, Hind Alsharhan, Paul R. Hillman, Leslie A. Dunnington, Hilde M. H. Braakman, Shane McKee, Angelica Moresco, Andrea-Diana Ignat, Ruth Newbury-Ecob, Guillaume Banneau, Olivier Patat, Jeffrey Kuerbitz, Susan Rzucidlo, Susan S. Sell, Patricia Gordon, Sarah Schuhmann, André Reis, Yosra Halleb, Radka Stoeva, Boris Keren, Zainab Al Masseri, Zeynep Tümer, Sophia Hammer-Hansen, Sofus Krüger Sølyst, Connolly G. Steigerwald, Nicolas J. Abreu, Helene Faust, Amica Müller-Nedebock, Frédéric Tran Mau-Them, Heinrich Sticht, Christiane Zweier

**Affiliations:** 1 Department of Human Genetics, Inselspital Bern, University of Bern, Bern, Switzerland; 2 Department for Biomedical Research (DBMR), University of Bern, Bern, Switzerland; 3 Center for Genomic Medicine, Parc Taulí Hospital Universitari, Institut d’Investigació i Innovació Parc Taulí (I3PT-CERCA), Universitat Autònoma de Barcelona, Sabadell, Spain; 4 Department of Molecular and Human Genetics, Baylor College of Medicine, Houston, TX USA; 5 Department of Neurosciences, Boystown National Research Hospital, Boystown, TX USA; 6 Section of Pediatric Neurology and Developmental Neuroscience, Department of Pediatrics, Baylor College of Medicine, Houston, TX USA; 7 The University of Kansas Health System, Westwood, KS USA; 8 Division of Child Neurology and Inherited Metabolic Diseases, Centre for Pediatrics and Adolescent Medicine, University Hospital Heidelberg, Heidelberg, Germany; 9 Division of Genetics, Department of Pediatrics, McMaster Children’s Hospital, Hamilton, ON Canada; 10 Division of Clinical and Metabolic Genetics, The Hospital for Sick Children, University of Toronto, Toronto, ON Canada; 11 Institute of Medical Genetics, University of Zurich, Zurich, Switzerland; 12 Department of Medical Genetics, CHU Nantes, Nantes, France; 13 Institute of Medical Genetics and Applied Genomics, University of Tübingen, Tübingen, Germany; 14 Center for Rare Diseases, University of Tübingen, Tübingen, Germany; 15 University Children’s Hospital, Klinikum Oldenburg, Department of Neuropediatrics, Oldenburg, Germany; 16 Mission Fullerton Genetics Center, Asheville, NC USA; 17 Department of Human Genetics, Radboud University Medical Center, Nijmegen, The Netherlands; 18 Center for Integrative Genomics, University of Lausanne, Lausanne, Switzerland; 19 Lahore College for Women University, Lahore, Pakistan; 20 Department of Medical Genetics, Le Havre Hospital, Le Havre, France; 21 Division of Medical Genetics, Department of Pediatrics, Weill Cornell Medicine, New York, NY USA; 22 Institute of Human Genetics, Klinikum rechts der Isar, Technical University of Munich, TUM School of Medicine and Health, Munich, Germany; 23 Department of Nephrology, Klinikum rechts der Isar, Technical University of Munich, TUM School of Medicine and Health, Munich, Germany; 24 GeneDx, Gaithersburg, MD USA; 25 Institute of Human Genetics, University of Bonn, School of Medicine and University Hospital Bonn, Bonn, Germany; 26 Department of Genetics, Université de Montréal, Sainte-Justine University Hospital, Montreal, Canada; 27 Medical Genetics Unit, Hospital Pediátrico de Coimbra, Unidade Local de Saúde de Coimbra, Coimbra, Portugal; 28 Centro de Diagnóstico Pré-natal, Unidade Local de Saúde de Coimbra, Coimbra, Portugal; 29 Department of Neurology, Boston Children’s Hospital and Harvard Medical School, Boston, MA USA; 30 Department of Pediatrics and Child Health, Dunedin School of Medicine, Otago University, Dunedin, New Zealand; 31 Center for Rare Disease Essen (Essener Zentrum für Seltene Erkrankungen-EZSE), Universitätsmedizin Essen, Essen, Germany; 32 Institut für Humangenetik, Universitätsklinikum Essen, Universität Duisburg-Essen, Essen, Germany; 33 Praxis für Humangenetik, Klinikum Bremen-Mitte, Bremen, Germany; 34 Department of Pathology and Molecular Medicine, McMaster University, Hamilton, ON Canada; 35 Kuwait Medical Genetics Centre, Ministry of Health, Sulaibikhat, Kuwait; 36 Department of Pediatrics, Health science center, College of Medicine, Kuwait University, P.O. Box 24923, Safat, Kuwait; 37 Department of Pediatrics, Division of Medical Genetics, McGovern Medical School at the University of Texas Health Science Center at Houston (UTHealth Houston) and Children’s Memorial Hermann Hospital, Houston, TX USA; 38 Department of Pediatric Neurology, Amalia Children’s Hospital, Radboud University Medical Center & Donders Institute for Brain, Cognition and Behavior, Nijmegen, The Netherlands; 39 Belfast HSC Trust, Northern Ireland Regional Genetics Service, Belfast, Northern Ireland; 40 Division of Clinical Genetics, Pediatric Department, Children’s Hospital, London Health Sciences Centre, Western University, London, ON Canada; 41 Clinical Genetics, University Hospitals Bristol, Southwell St, Bristol, UK; 42 Department of Medical Genetics, Toulouse University Hospital, Toulouse, France; 43 Cain Pediatric Neurology Research Foundation Laboratories, Jan and Dan Duncan Neurological Research Institute, Texas Children’s Hospital, Houston, TX USA; 44 Penn State Health Children’s Hospital, Department of Pediatrics, Division of Human Genetics, Hershey, PA USA; 45 Institute of Human Genetics, Universitätsklinikum Erlangen, Friedrich-Alexander-Universität Erlangen-Nürnberg, Erlangen, Germany; 46 Centre for Rare Diseases Erlangen (ZSEER), Erlangen, Germany; 47 Le Mans Hospital, Department of Medical Genetics, Le Mans, France; 48 Department of Genetics, Assistance Publique - Hôpitaux de Paris, Hôpital Pitié-Salpêtrière, Paris, France; 49 Department of Pediatrics, Medical Genetics Unit, Qatif Central Hospital, Eastern Health Cluster, Dammam, Saudi Arabia; 50 Kennedy Center, Department of Clinical Genetics, Copenhagen University Hospital, Rigshospitalet, Copenhagen, Denmark; 51 Department of Clinical Medicine, Faculty of Health and Medical Sciences, University of Copenhagen, Copenhagen, Denmark; 52 Department of Clinical Genetics, Copenhagen University Hospital, Rigshospitalet, Copenhagen, Denmark; 53 Division of Neurogenetics, Department of Neurology, NYU Grossman School of Medicine, New York, NY USA; 54 Institute of Human Genetics, University of Leipzig Medical Center, Leipzig, Germany; 55 Unité Fonctionnelle Innovation en Diagnostic Génomique des Maladies Rares, CHU Dijon Bourgogne, Dijon, France; 56 Génétique des Anomalies Du Développement, INSERM 123, Université de Bourgogne, Dijon, France; 57 Institut für Biochemie, Friedrich-Alexander-Universität Erlangen-Nürnberg, Erlangen, Germany

**Keywords:** Genetics research, Autism spectrum disorders

## Abstract

While mostly de novo truncating variants in *SCAF4* were recently identified in 18 individuals with variable neurodevelopmental phenotypes, knowledge on the molecular and clinical spectrum is still limited. We assembled data on 50 novel individuals with *SCAF4* variants ascertained via GeneMatcher and personal communication. With detailed evaluation of clinical data, in silico predictions and structural modeling, we further characterized the molecular and clinical spectrum of the autosomal dominant *SCAF4*-associated neurodevelopmental disorder. The molecular spectrum comprises 25 truncating, eight splice-site and five missense variants. While all other truncating variants were classified as pathogenic/likely pathogenic, significance of one C-terminal truncating variant, one splice-site variant and the missense variants remained unclear. Three missense variants in the CTD-interacting domain of SCAF4 were predicted to destabilize the domain. Twenty-three variants occurred de novo, and variants were inherited in 13 cases. Frequent clinical findings were mild developmental delay with speech impairment, seizures, and skeletal abnormalities such as clubfoot, scoliosis or hip dysplasia. Cognitive abilities ranged from normal IQ to severe intellectual disability (ID), with borderline to mild ID in the majority of individuals. Our study confirms the role of *SCAF4* variants in neurodevelopmental disorders and further delineates the associated clinical phenotype.

## Introduction

Both initiation and proper termination of transcription are crucial for cell function and survival [1, 2]. While transcription initiation via RNA polymerase II has already been explored in depth, the regulation of elongation and mRNA termination are less well understood. One factor involved in termination of RNA polymerase II mediated transcription is the SR-related CTD-associated factor 4 (SCAF4) (MIM: 616023). *SCAF4* in its longest isoform consists of 20 exons and encodes a splicing factor containing the well conserved N-terminal CTD-interacting domain (CID), as well as an arginine/serine rich domain, and an RNA binding motif [3]. With its CTD-interacting domain, SCAF4 binds to the C-terminal domain of the largest subunit of RNA polymerase II, encoded by *POLR2A*, another gene implicated in a neurodevelopmental disorder [4–6]. Together with SCAF8, SCAF4 is responsible for preventing early mRNA termination by suppressing the use of alternative Poly-A sites during transcription and also secures termination at the right site [7]. Heterozygous alterations of *SCAF4* have been shown to lead to deregulation of more than 9000 genes, including some involved in gene regulation and RNA processing [8].

Recently, variants in *SCAF4* have been reported to cause a neurodevelopmental disorder (NDD) (Fliedner-Zweier syndrome, MIM#620511) [8–10]. In 2020, eight truncating, two splice-site and one missense variant were reported in 11 individuals with variable neurodevelopmental phenotypes, including mild intellectual disability, seizures, and various organ or skeletal anomalies [8]. Since then, only seven additional individuals carrying a *SCAF4* variant and with intellectual disability, epilepsy, and cardiac defects have been reported [9–11].

**Fig. 1:**
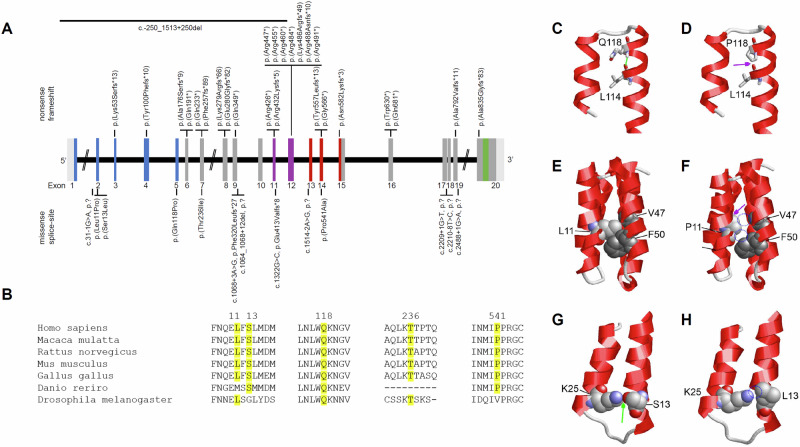
*SCAF4* variants in individuals with Fliedner-Zweier syndrome. **A** Exon structure of *SCAF4* (NM_020706.2, NP_065757.1) with newly identified variants and a larger intragenic deletion. Domains color coded according to ensemble, white for non-coding exonic regions, gray for coding exons and color for annotated domains (blue: CTD-interacting domain (CID), purple: Ser/Arg rich domain (SR), red: RNA binding motif (RRM), green: Pro/Gln rich domain (PQ)) [34]. **B** Conservation of missense variants from *Homo sapiens* to *Drosophila melanogaster*. All variants are fully conserved in tetrapods. Variant positions are highlighted in yellow. Alignment was performed with Clustalw2 [35, 36] with the following reference sequences: *Homo sapiens*: NP_065757.1, *Macaca mulatta*: XP_014988521.2, *Rattus norvegicus*: NP_001032424.2, *Mus musculus*: NP_001404016.1, *Gallus gallus*: NP_001012840.1, *Danio rerio*: NP_001373157.1, *Drosophila melanogaster*: NP_001097394.1. Effect of the p.(Gln118Pro), p.(Leu11Pro) and p.(Ser13Leu) exchanges on the structure of the CTD-interacting domain. **C** In wt-SCAF4, the amide hydrogen of Q118 forms a hydrogen bond with L114 (green line). **D** In the Q118P variant, this hydrogen bond cannot be formed because proline lacks an amide proton. Instead, steric clashes occur between the cyclic sidechain of P118 and L114 (magenta arrow), which are expected to decrease helix stability. **E** L11 of wildtype SCAF4 forms hydrophobic sidechain interactions with V47 and F50 (shown in gray). **F** In the L11P variant, these hydrophobic interactions cannot be formed by the cyclic sidechain of the proline residue, which is predicted to destabilize the structure of the CTD-interacting domain. Lacking hydrophobic interactions in the variant are highlighted by blue dotted circles. In addition, the cyclic proline sidechain induces steric clashes within the helix (magenta arrow), which further decrease protein stability. **G** S13 of wildtype SCAF4 forms a sidechain hydrogen bond with K25. Both residues are shown in space-filled presentation and colored according to the atom types; the hydrogen bond between the sidechain oxygen (red) of S13 and the sidechain nitrogen (blue) of K25 is indicated by a green arrow. **H** In the p.(Ser13Leu) variant, this hydrogen bond cannot be formed by the nonpolar sidechain of L13, which is predicted to destabilize the structure of the CTD-interacting domain. The site of the lacking hydrogen bond is highlighted by a blue dotted circle.

We now have assembled a cohort of 50  further affected individuals with variants in *SCAF4* (Fig. 1A) and thus considerably broaden the molecular and clinical spectrum of the *SCAF4*-associated NDD.

## Materials and methods

### Molecular and clinical data

Affected individuals were ascertained through international collaboration using GeneMatcher [12] and personal communication. Clinical data were collected using a standardized excel template (Supplemental Table S1). Testing by (trio) exome or genome sequencing was either performed in a diagnostic setting, not requiring a specific ethical approval, or in a research setting with ethical approval from institutional review boards of the respective centers (Commission d'éthique de la recherché canton Vaud, Lahore College for Women University, Kantonale Ethikkomission Zürich, Kantonale Ethikkommission Bern, Technical University of Munich, Harvard (IRB-P00032816), Supplemental Table S1). Segregation analysis for familial variants was performed by Sanger sequencing or by quantitative RT-PCR (Supplemental Table S1). Variants were classified according to ACMG criteria [13] plus current ClinGen sequence variant interpretation recommendations (PS2/PM6 version 1.1; splicing recommendations [14]). Information on additional variants and the phenotypes of the affected individuals is detailed in Supplemental Table S2. Consent for publication of genetic and clinical data, as well as photographs was obtained from the individuals, their parents, or legal guardians, respectively. The study complied with the principles set out in the Declaration of Helsinki.

### In vitro splice assay

To investigate a potential splice effect of the variant c.2210-8 T > C, an exon trapping mini-gene assay was performed. The sequence of exons 17 and 18 (primer pair 1, F: tgaatagaagttaactaccacctgt, R: attaattatctcccggtcaacattc) or exon 18 alone (primer pair 2, F: ctttagagcctgcgatggag, R: attaattatctcccggtcaacattc) of *SCAF4* and the surrounding introns (primer pair 1: 5’ intron: 160 bp, 3’intron: 119 bp, primer pair 2: 5’ intron: 58 bp, 3’intron: 119 bp) were cloned into the empty pSPL3 vector (Thermo Fisher Scientific). The regions were amplified from genomic DNA, and site directed mutagenesis (Agilent) was used to introduce the specific variant into the plasmid. Constructs pSPL3-SCAF4_WT_1, pSPL3-SCAF4_Var_1, pSPL3-SCAF4_WT_2 and pSPL3-SCAF4_Var_2 were transiently transfected into HEK293 cells using the jetPrime transfection system (Polyplus Transfection). 48 h post transfection, RNA was isolated from the cells using the RNeasy Plus Mini Kit (Qiagen). cDNA was reversely transcribed using SuperScript II (Thermo Fisher Scientific). RT-PCR was performed using standard primers (F: TCTGAGTCACCTGGACAACC, R: ATCTCAGTGGTATTTGTGAGC) to amplify the product transcribed from the vector. Resulting products were analyzed using gel electrophoresis and by sequencing. Sequences were aligned and depicted with Benchling biology software (www.benchling.com).

### Structural modeling

The effect of the p.(Leu11Pro), p.(Ser13Leu) and p.(Gln118Pro) missense variants was modeled using the crystal structure of the SCAF4 CTD-interacting domain (PDB:6XKB) [15]. Variants were modeled with SwissModel [16], and RasMol [17] was used for structure analysis and visualization.

## Results

### *SCAF4* molecular spectrum

We assembled 50 individuals from 41 families with 39 different, novel variants in *SCAF4* (Fig. 1A). Twenty-three of these variants occurred de novo, in 13 cases the variant was inherited, and in 14 individuals parents were not available for testing. In individual 15, the variant was detected as mosaic in 8% of the peripheral blood cells.

Identified variants included an intragenic deletion of exons 1–12, 25 truncating (nonsense or frameshift) variants, eight splice-site variants and five missense variants. Aberrant splicing was confirmed in mRNA for c.1068+3 A > G (individual 12) and for c.1322 G > C (individual 16) in the respective centers (Supplemental Table S1). In silico analysis of the remaining six splice-site variants using SpliceAI [18] and Pangolin [19] scores predicted a likely splice-site alteration for five variants in individuals 1, 13, 32, 39 and 42 (c.31-1 G > A, c.1064_1068+12del, c.1514-2 A > G, c.2209+1 G > T, and c.2488+1 G > A, respectively). The variant c.2210-8 T > C in individual 40 was predicted to not have an impact on splicing (Supplemental Table S4). In accordance, testing this variant in an in vitro splice assay did not indicate aberrant splicing (Supplemental Fig. S1). Truncating, including splice-site variants were distributed ubiquitously over the gene/protein, thus being compatible with a general loss-of-function (LoF) effect or haploinsufficiency (Fig. 1A). Of note, one of the protein truncating variants was located in the ultimate exon and was predicted to escape nonsense mediated mRNA decay.

Three of the five missense variants (p.(Leu11Pro), p.(Ser13Leu), p.(Gln118Pro)) localized to the CTD-interacting domain, while a fourth (p.(Pro541Ala)) resided in the RNA recognition motif (RRM). All five missense variants were predicted to be deleterious by at least six variant effect prediction scores (REVEL [20], M-CAP [21], CADD [22], GERP + +  [23], SIFT [24], MutationTaster [25], and POLYPHEN [26]) (Supplemental Table S3), and affected amino acid residues were highly conserved (Fig. 1B). *SCAF4* was predicted to be highly intolerant towards LoF variants (pLI = 1, observed/expected = 0.19, LOEUF = 0.29) [27], while this was not the case for missense variants (*z* = 1.48, o/e = 0.89). Structural modeling for the three missense variants in the CTD-interacting domain indicated that each of them significantly disrupt the domain structure (Fig. 1C–H). In two of the variants (p.(Leu11Pro), p.(Gln118Pro)) a proline emerged within an α-helix. The cyclic structure of the proline sidechain induced steric clashes within the helix, which is expected to decrease domain stability (Fig. 1C–F). For two of the variants, destabilization between adjacent helices is caused through the loss of hydrophobic (p.(Leu11Pro); Fig. 1E, F) or polar sidechain interactions (p.(Ser13Leu); Fig. 1G, H). Structural destabilization of the CTD-interacting domain has also been recently reported for a p.(Cys54Arg) variant [11]. Since the CTD-interacting domain interacts with the C-terminal heptapeptide repeat domain (CTD) of RNA polymerase II [7, 15], it is expected that disruption of the CTD-interacting domain structure will also strongly impair the interaction between SCAF4 and the polymerase.

Variants assembled in this study were not or only very infrequently (one to two allele counts) listed in the gnomAD database (v4.0) [28], detailed information can be found in supplemental Table S1. All but two (I40 (c.2210-8 T > C) and I43 (p.(Ala835Glyfs*83))) of the truncating and splice-site variants were classified as likely pathogenic or pathogenic according to current ACMG criteria [13] (Supplemental Table S1). Though there is some evidence for pathogenicity via in silico prediction, de novo occurrence and/or structural modeling also for the remaining variants, they were currently classified as variants of uncertain significance. Classification and applied criteria are detailed in Supplemental Table S1.

In 19 individuals, additional variants, either (likely) pathogenic or of uncertain significance in other disease genes were identified (Supplemental Table S2), of which at least some might contribute to the respective individual’s phenotype. For example, individual 6 harbored a maternally inherited variant in *CLCN1* (MIM: 160800) (Myotonia congenita Thomsen), which might explain sleeping difficulties and muscular hypertonia [29]. A 9q33 deletion was found in individual 7, which is also associated with intellectual disability, epilepsy, ambiguous genitalia and nail patella syndrome [30]. Also, the inherited variant in *TLK2* (MIM: 618050) in individual 50 might contribute to her intellectual disability and hypotonia [31].

### Clinical spectrum

**Fig. 2:**
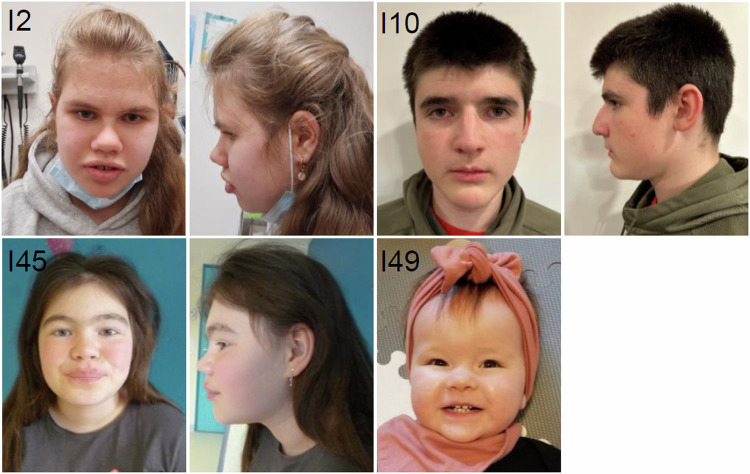
Clinical photographs of affected individuals. Note the broad nasal bridge in I2, I10, and I45.

Developmental delay and/or intellectual disability (ID) were reported in all but three individuals in this study, who were tested because of epilepsy or muscular hypotonia and behavioral issues. Therefore, penetrance of *SCAF4*-associated phenotypes appears to be high but clinical presentation is variable. Cognitive impairment ranged from learning difficulties or mild ID in 36 individuals to severe ID in three individuals. In the familial cases with available data, six of eight transmitting parents were reported to have had or still have learning difficulties or mild behavioral abnormalities. Only two mothers were reported to be unremarkable regarding developmental delay or learning issues. Speech development appeared to be more severely affected than motor development, with impaired speech reported in 36 individuals and delayed walking reported only in ten individuals, respectively. Behavioral or psychiatric disturbances were frequently observed (40 individuals) and included aggression, attention deficit hyperactivity disorder, autism spectrum disorder and tics. Sleeping difficulties were reported in 16 individuals and feeding difficulties in 10. Seizures with an age of onset between 9 months and 13 years occurred in 20 individuals. Seizure types were both focal and generalized, and EEG anomalies included polymorphic wave patterns and multifocal spike waves. MRIs were performed in 27 cases and revealed non-specific abnormalities in eight of them such as periventricular leukomalacia and prominent ventricles, as well as Chiari malformation. Variable eye or skeletal anomalies were reported in 16 and 23 individuals each. Other abnormalities were infrequent and included muscular hypo- or hypertonia(14x) postnatal micro-(9x) or macrocephaly, (1x) urogenital, (11x) renal (4x) and cardiac anomalies (3x). Facial dysmorphism were reported in most of the individuals but were rather non-specific (Fig. 2). Individual 15 with the mosaic truncating variant presented with developmental delay in early childhood, some behavioral issues in infancy and a multicystic left kidney, a rather mild but similar presentation compared to individuals with germline variants.

In total, detailed clinical information on seven adults ( > 18 years) was available. Though data is not sufficient to draw detailed conclusions on natural history, mild developmental delay and mild cognitive presentation in infancy seems to correlate well with a mild manifestation in adulthood in terms of learning difficulties but capacities for a normal job and a family. No major, late-onset or progressive health issues were reported in any of the adult individuals.

The clinical phenotype associated with missense variants was not distinguishable from that associated with truncating variants. More detailed information on the clinical spectrum can be found in Table 1 and in Supplemental Table 1.

**Table 1:** Clinical summary.

Variant type	Truncating, this cohort, *n* = 45	Truncating, previously published, *n* = 16 ^8–11^	Missense, this cohort, *n* = 5	Missense previously published, *n* = 2 ^8,11^	Total
ID/DD				1/1	
*mild*	32/44	11/15	4/5		47/64 (73%)
*moderate*	7/44	3/15	1/5		11/64 (17%)
*severe*	3/44	1/15	0/5		4/64 (6%)
Seizures	18/43	11/15	2/5	2/2	33/65 (51%)
Behavioral/psychiatric disturbances	36/43	9/10	4/5	1/1	50/59 (85%)
Hypotonia	6/40	4/8	3/5		13/53 (25%)
Hypertonia	4/40	1/8	1/5		6/53 (11%)
Macrocephaly	1/38		0/3	1/1	2/42 (5%)
Microcephaly	9/38	1/1	0/3		10/42 (24%)
Eye abnormalities	16/41	1/1	0/5		17/47 (36%)
Facial dysmorphism	28/42	2/2	1/5		31/49 (63%)
Short stature	9/42		1/5		10/47 (21%)
Sleeping difficulties	15/40	1/1	1/5		17/46 (37%)
Feeding difficulties	9/37	1/1	1/5		11/43 (26%)
Urogenital anomalies	10/41	4/8	1/5		15/54 (28%)
Renal anomalies	4/34	4/6	0/5		8/45 (18%)
Skeletal anomalies	21/41	8/10	2/5	1/1	32/57 (56%)
Cardiac anomalies	3/36	4/6	0/5		7/47 (15%)

*ID* intellectual disability, *DD* developmental delay;

## Discussion

By assembling 50 additional individuals, we further delineate the molecular and clinical spectrum of *SCAF4*-associated NDD (MIM#620511).

The most prominent feature is developmental delay and intellectual disability, mostly in the borderline to mild range, and language and speech delay. Mild manifestation in childhood seems to go in hand with a rather mild and favorable outcome in adulthood, many of the adult individuals presenting with learning difficulties to some degree but normal jobs and families. Also, behavioral issues such as attention deficit hyperactivity disorder, autistic features and aggressivity are frequently reported. While seizures have been previously reported in 60–100% of affected individuals [8–11], they occur only in 42% of individuals in this cohort, adding up to a total frequency of 51% in all reported individuals. An apparently higher prevalence in the initial reports might be due to a bias in the previously much smaller group. Seizures have been previously described as focal in affected individuals [10] and as reflex seizures in a zebrafish model [32]. With more cases known, the spectrum now seems to be more variable, including generalized epilepsy in this cohort and another report [11]. Other clinical aspects less commonly seen in this, larger cohort are hypotonia, cardiac, and renal anomalies, which were previously described in up to 67% of the individuals and now only appear in 7–20% (detailed information can be found in Table 1) [8–11]. Also for this observation a previous bias due to previously smaller case numbers is likely. Further frequent aspects of *SCAF4*-associated NDD are non-specific facial dysmorphism and skeletal abnormalities, while other morphological or organ abnormalities are noted only infrequently. This characterizes the *SCAF4*-associated phenotype as a rather mild and non-specific neurodevelopmental disorder with variable clinical expression and high, though not complete, penetrance. Due to a certain prevalence of cardiac, renal, urogenital and ophthalmological anomalies, a specific check for such abnormalities might be considered at time of diagnosis.

As the majority of identified disease-related variants in *SCAF4* are truncating, this led to the prediction of haploinsufficiency as the most likely disease mechanism [8]. In accordance, most variants assembled in the current cohort are truncating as well. Nevertheless, in total seven missense variants were reported, five in this and two in previous studies [8, 11], most of them de novo and residing in functional domains, thus predicted to possibly also result in loss of protein function.

Interestingly, in our assembled cohort there was a relatively large number of inherited *SCAF4* variants (13 cases). Most variant carrying parents or siblings in these familial cases also presented with similar neurodevelopmental phenotypes. This is in accordance with *SCAF4*-associated NDD being rather mild regarding the cognitive phenotype but might provide some challenges for variant filtering and interpretation in trio exome sequencing approaches.

The rather non-specific, mild phenotype might also complicate interpretation of additional variants in other disease related genes. These additional variants might be (re)classified as (likely) benign or deleterious in the future [33], the latter thus contributing to the variable phenotype, possibly within an oligogenic context.

To conclude, through assembling a larger cohort of individuals with variants in *SCAF4*, we were able to further delineate the phenotype of the *SCAF4*-associated NDD. Our findings support haploinsufficiency as the most likely pathomechanism.

## Supplementary information

Supplementary Table S1

Supplementary Table S2

Supplementary data

## Data Availability

Data supporting the findings of this study are provided in the manuscript or the supplementary material or is available from the corresponding author upon request.
