# Supplementary material for: Further delineation of the SCAF4-associated neurodevelopmental disorder
Source: Eur J Hum Genet. 2024 Dec 12;33(5):588–94. doi: 10.1038/s41431-024-01760-2 (PMC12048650; doi:10.1038/s41431-024-01760-2)
Supplement: Supplementary file 3 — Supplementary data [file 41431_2024_1760_MOESM3_ESM.pdf]

Supplementary material

Supplemental Figures

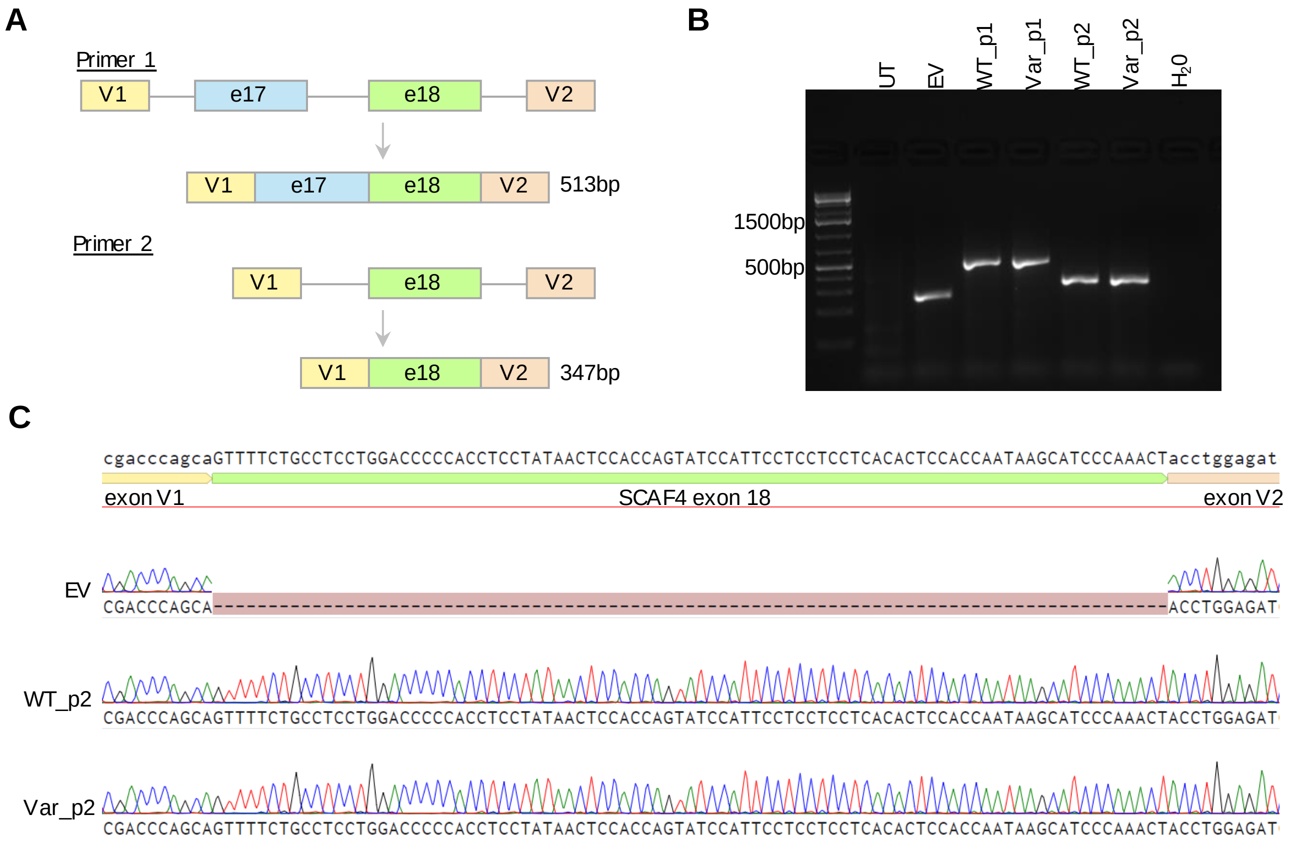

Figure S1 Testing of splice-site variant c.2210-8T>C in an in vitro splice assay. A. Schematic overview of assay design and expected RT-PCR products. **B.** No alterations of splicing due to variant c.2210-8T>C was observed in RT-PCR of *in vitro* splice assay for two different tested constructs on agarose gel. **C.** Sanger sequencing confirmed lack of effect on splicing of variant c.2210-8T>C. Sequences were aligned and depicted with Benchling biology software (www.benchling.com). Abbreviations: V1- 5' exon in pSPL3 vector, V2 – 3' exon in pSPL3 vector, e17/18 – SCAF4 exon

17/18, UT – untransfected cells, EV – empty pSPL3 vector, WT_p1/2 – pSPL3 with product of primer pair 1/2 with WT sequence, Var_p1/2 – pSPL3 with product of primer pair 1/2 with variant-containing sequence.

Supplemental Tables

Table S1 detailed variant and clinical information of 50 individuals reported in this study (excel file).

Table S2 additionally identified variants and phenotypes of affected individuals (excel file).

Table S3. *In silico* prediction of missense variants in *SCAF4*

**Individual**	**46**	**47**	**48**	**49**	**50**
**Variant (NC_000021.8 NM_020706.2)**	g.33078669A>G c.32T>C p.(Leu11Pro)	g.33078663G>A c.38C>T p.(Ser13Leu)	g. 33074661T>G c.353A>C p.(Gln118Pro)	g.33073378G>A c.707C>T p.(Thr236Ile)	g.33064237G>C c.1621C>G p.(Pro541Ala)
**REVEL**	0.90852	0.73372	0.93931	0.51265	0.7278
**M-CAP**	0.88237	0.67421	0.79512	0.35345	0.46453
**CADD**	25.4	23.1	26.1	25.6	27.2
**GERP++**	5.92	5.92	5.69	6.07	5.33
**SIFT**	D(0)	D(0)	D(0)	D(0)	D(0)
**Mutation taster**	D	D	D	D	D
**POLYPHEN**	probably_damaging(0.985)	benign(0.314)	probably_damaging(0.996)	possibly_damaging(0.468)	probably_damaging(0.998)
**gnomAD frequency**	0	1	0	0	0

Table S4. *In silico* prediction of splice-site variants in *SCAF4*

**Individual**	**1**	**12**	**13**	**32**	**39**	**40**	**42**
**Variant (NC_000021.8 NM_020706.2)**	g.33078671C>T c.31-1G>A p.?	g.33068423T>C c.1068+3A>G p.Phe320Leufs*27	g.33068414_33068430del c.1064_1068+12del p.?	g.33064764T>C c.1514-2A>G p.?	g.33057880C>A c.2209+1G>T p.?	g.33057805A>G c.2210-8T>C p.?	g.33057361C>T c.2488+G>A p.?
**Splice AI**	Acceptor loss 0.99 Acceptor gain 0.75	Donor loss 0.03 Donor gain 0.13	Donor loss 1.00 Donor gain 0.30	Acceptor loss 1.00 Acceptor gain 1.00	Donor loss 0.99 Donor gain 0.01	Acceptor/Donor loss 0.00 Acceptor/Donor gain 0.00	Donor loss 0.99 Donor gain 0.01
**Pangolin**	Splice loss 0.89 Splice gain 0.65	Splice loss 0.25 Splice gain 0.02	Splice loss 0.96 Splice gain 0.13	Splice loss 0.86 Splice gain 0.88	Splice loss 0.86 Splice gain 0.02	Splice loss 0.00 Splice gain 0.00	Splice loss 0.89 Splice gain 0.22
**gnomAD frequency**	0	0	0	0	0	0	0
